# TEI-friendly annotation scheme for medieval named entities: a case on a Spanish medieval corpus

**DOI:** 10.1007/s10579-020-09516-2

**Published:** 2021-02-27

**Authors:** Elena Álvarez-Mellado, María Luisa Díez-Platas, Pablo Ruiz-Fabo, Helena Bermúdez, Salvador Ros, Elena González-Blanco

**Affiliations:** 1 Digital Humanities Innovation Lab (LINHD), School of Computer Science, UNED University, Madrid, Spain; 2 CoverWallet, Madrid, Spain

**Keywords:** Named-entity annotation, Annotation scheme, Historical NER, Medieval named entities, Medieval Spanish corpus

## Abstract

Medieval documents are a rich source of historical data. Performing named-entity recognition (NER) on this genre of texts can provide us with valuable historical evidence. However, traditional NER categories and schemes are usually designed with modern documents in mind (i.e. journalistic text) and the general-domain NER annotation schemes fail to capture the nature of medieval entities. In this paper we explore the challenges of performing named-entity annotation on a corpus of Spanish medieval documents: we discuss the mismatches that arise when applying traditional NER categories to a corpus of Spanish medieval documents and we propose a novel humanist-friendly TEI-compliant annotation scheme and guidelines intended to capture the particular nature of medieval entities.

## Introduction

Written text is the primary means of access to the past. Political treaties, legal records, commercial transactions, notarial deeds, chronicles: these historical documents constitute the main piece of evidence of historical events. Our understanding of historical developments relies heavily on the textual information they contain. However, this historical data is frequently sparse and fragmentary, scattered among many documents or even unmanageable. As valuable as it may be, data is hardly of any use if it cannot be retrieved effectively. That is why applying Information Extraction (IE) techniques to historical documents once it has been digitalized and transcribed properly is particularly fruitful: automatically extracting the names of places, people or organizations mentioned on historical documents can provide us with valuable historical evidence that otherwise would be extremely cumbersome to obtain or could even go unnoticed under a pile of documents.

*Named entity* is a widely used term in Natural Language Processing (NLP) that refers precisely to textual information units like person names, location names, organization names, etc. The task of identifying these entities is known as Named Entity Recognition (NER). NER is one of the main subtasks of IE (Grishman and Sundheim 1996) and a key step in text analysis, as it provides information on what and who are mentioned in a given text (Nadeau and Sekine 2007). Consequently, NER has steadily remained a hot topic in NLP for the last twenty years.

NLP has dealt extensively with the mechanisms and difficulties that NER poses (Tjong Kim Sang and De Meulder 2003). Numerous NER systems have been proposed, traditionally trained on easily available large general-domain corpora written in resource-rich languages, such as newspaper articles or newswire (Desmet and Hoste 2014). However, general-domain techniques tend to perform poorly when used for unseen genres (Plank 2016; Poibeau and Kosseim 2001). Historical documents present remarkable differences compared to general-domain texts. In fact, historical language has been described as a type of under-resourced language (Pettersson et al. 2013) that is highly non-canonical both in terms of content (genre and domain) and form (linguistic variation, lack of orthographic standardization) (Sánchez-Marco et al. 2011). As a result, NER annotation schemes designed with modern general-domain text in mind fail to capture the nature of historical named entities.

Although historical documents have traditionally been neglected in terms of text analysis in favor of more accessible and industry-appealing fields (such as the biomedical, social or journalistic domain), a growing number of interdisciplinary projects born within the field of Digital Humanities (which deals with the digitization, preservation and study of historical documents) are promoting a data-driven approach to historical texts (Piotrowski 2012). Previous work has shown the potential of applying and adapting Digital Humanities standards (such as TEI-XML, the standard for the digital encoding of texts in the humanities) to perform information extraction tasks on historical texts. For example, initiatives like the Pleiaides Gazetteer or Pelagios Commons (Isaksen et al. 2014) localize places mentioned in documents from the Antiquity and collect them in an open and interoperable format. And Maraoui et al. (2018) adapted TEI-XML standards to encode Arabic person names in order to annotate the Hadith corpora, a collection of narrations of the deeds of Prophet Muhammad that are relevant for understanding the Qur’an jurisprudence. However, the medieval world still remains mostly unexplored in terms of NER exploitation, in spite of the attractiveness of its vast textual tradition.

Annotating is the process of enriching a collection of text by adding linguistic and interpretative information to it (Hovy and Lavid 2010). Annotation is a fundamental step towards the development and evaluation of a NER system: human-annotated corpora provide us with valuable data that can serve both as training material for machine learning approaches as well as a gold standard against which new algorithms and techniques will be evaluated, Pustejovsky and Stubbs (2012). Consequently, it is key to have a set of shared criteria to follow that describe the nature and format of the annotation process, i.e. an annotation scheme. Annotation schemes contribute to narrowing down the linguistic phenomena to be tackled and produce more reliable and coherent annotations (Bayerl et al. 2003). This is particularly interesting when dealing with highly non-canonical texts, such as historical language, where non-standardized spelling and format can make reaching an agreed consensus on which text sequences should be annotated particularly elusive.

In this paper we present a novel humanist-friendly annotation scheme and annotation guidelines specifically designed to capture the particular nature of medieval named entities. This annotation proposal is an adaptation of traditional name entity classifications tailored to suit the medieval domain and to facilitate the application of NER techniques (which are far from optimal to reflect the essence of medieval reality) to medieval documents. This annotation scheme complies with XML-TEI markup, the XML standard for annotation and representation of texts among digital humanists.

The annotation scheme we present in this paper is conceived for the identification of named entities in Medieval Spanish texts. The motivation to undertake this annotation task was to provide support (both in terms of annotation scheme and gold standard creation) to HisMeTag (Hispanic Medieval Tagger) (Díez Platas et al. 2017, 2020), a NER tool that was developed in collaboration with Pelagios project^1^, whose goal is to supply linked open data methods to relate and explore historical places. The purpose of HisMeTag is to support digital humanists in the analysis of medieval texts by detecting, annotating and geolocalizing historical places on a given document through contextual information. The F1 score obtained by this tool ranges between 0,63 and 0,89 depending on the time period of the document (Díez Platas et al. 2017).

This paper is organized as follows: in Sect. 2 we explain which named entity categories have been considered for the present scheme; in Sect. 3 we describe the annotation scheme itself (both the annotation format and the tagset); in Sect. 4 we provide the annotation guidelines that should be followed when applying the annotation scheme to a medieval corpus and how to deal with problematic cases that can occur in medieval documents; Sect. 5 contains the application of the scheme and guidelines to annotate a Spanish corpus of medieval documents along with the obtained results and error analysis; finally, Sect. 6 contains the conclusions and future work.

## Annotation framework

In this section we introduce the types and idiosyncrasies of named entities in the medieval domain.

### Rationale

Named entities are usually classified according to the type of entity they refer to (such as person, location, etc). These categories are highly domain-dependent: while a journalistic NER scheme will be concerned with categories such as places or people, a biomedical NER scheme will deal with categories such as molecules or drugs. Therefore, it is key to define the set of NER categories and tailor it to the text domain that will be annotated.

In order to assess the need for tailored-made guidelines for the annotation of medieval named entities, several examples of medieval documents in Spanish were inspected, along with some general domain guidelines such as ACE guidelines (Linguistic 2005), MUC-7 guidelines (Chinchor 1998) and the Extended Named Entity Hierarchy (Sekine 2003). Named entity annotation on Medieval Spanish texts presents three specific challenges:

(i) The diversity of orthographic norm (Sánchez-Marco et al. 2011).
(ii) The complexity of morphosyntatic structures that can appear with proper proper nouns during this time period (with named entities occurring inside comprehensive structures that can include geographical origin or linage of the person being mentioned: *rey Alfonso de Castilla*, “King Alfonso of Castile”). These patterns create a set of dependencies between the entity and its joined attributes that the annotation scheme has to account for.
(iii) The profusion of person entities that are mentioned exclusively either through a nickname or through their social role within society (with no explicit person name whatsoever, for example: *El Cid*). The use of nicknames and role names as a way to refer to someone is by no means exclusive of the medieval domain and, in fact, can be found in other general-domain texts. However, the abundance of this phenomenon in medieval documents is extremely high: nicknames and role names may appear in modern texts along with the actual name of the person being mentioned. In the medieval domain, however, nicknames and role names are frequently the only way in which a person is referred to: therefore, not considering role names or nicknames as named entities would mean missing a great amount of entities appearances that will not be referred to in any other way in the text.

### Named-entity categories

In this section, we will describe the categories that have been considered for our annotation scheme. The proposed annotation scheme considers five general named-entity categories: **person names**, **location names**, **organization names**, **role names** and **miscellanea**. Person, location and organization names are inherited categories from the MUC 7 guidelines (Chinchor 1998). Role name is a tailor-made category to annotate medieval titles, positions and social relationships; this category is unprecedented in named entity categories and, to the best of our knowledge, is an original contribution of this work. Finally, miscellanea is an open-ended category that seeks to cover any historically-relevant named entity that does not fit into any of the other categories and that the annotators could decide that needed to be annotated (a precedent of this category can be found on Sekine et al. (2002) under the Name_Other category). The primary objective of this annotation project is to annotate places and people mentioned in medieval documents. Consequently, other entities (such as events, quantities, etc) were considered beyond the scope of the annotation project. The nature and scope of the five named-entity categories considered in this work are as follows (see Table 1 for comparative examples):

- **Person names** are proper names that refer to a person (e.g. *Alexander the Great*). When dealing with medieval documents, person names should be expected to refer to kings and queens, knights, nobility, gods and other mythological figures. Person names can be first names, surnames or a combination of both. Nicknames and aliases (which were quite frequent in medieval society and even work as official names on legal documents) will also be considered a type of person name. Both real people and fictitious characters will be considered as a person entity. Sekine et al. (2002) classify fictitious characters as a product rather than a person (what are fictitious characters but a type of mental human production?). However, this distinction between reality and fiction becomes rather blurred when dealing with medieval chronicles: historical accounts of true events are frequently sprinkled with mythical characters and legends. Therefore, no NER category distinction will be made between historical figures and literary characters within the medieval domain. Likewise, gods, deities and religious figures names (including biblical nicknames and divine aliases) will also be considered as a type of person entity.
- **Location names** refer to any location, including geopolitical units (countries, regions, towns, kingdoms etc; e. g. *Constantinople*, *Kingdom of León*), geographical names (mountains, rivers, landforms in general; e.g. *Mediterranean Sea*) or facilities (buildings and monuments, like castles, bridges or monasteries; e.g. *Church of Santa Gadea*). Generally speaking, any geolocalizable place (i.e. any given location that can be tracked on a map) will be considered a location entity^2^. Although religious and mythical places are not geolocalizable, they will also be considered a special type of location name (e.g. *Garden of Eden*, *Paradise* or *Styx river*).
- **Organization names** are associations, institutions or any other group of people. While in the journalistic domain organization entities tend to be companies, sports team or music bands, in medieval documents such associations are normally religious orders, armies or governmental institutions (e.g. *Order of St. James*, *Aragonese Courts*). Following (Sekine 2003), the organization category will be considered in a broad sense and will be applied to any coherent group of people that share a name and a sense of unity or cause, like names of peoples or religious and ethnic communities (e.g. *the Greeks*, *the Trojans*, *the Moors*, *the Jews* or *the Christians*).
- **Role names** are names that refer to medieval positions, nobility titles, professions or family relations. In broad terms, role names are any named relationships or states that establish a type of bond between two or more individuals, an individual and a group or an individual and a geopolitical entity. These relationships can work *de facto* as a way to identify someone within the medieval society and include titles like “the queen” or “the Pope” as well as professions like “the baker” or “the priest” and relations like being someone’s parent or someone’s heir. Role names (such as “King of Castilla”) enable the establishment of relationships at three levels: firstly, between the person and their role; secondly, between the role and the place upon which this role has relevance; and finally, by inference, between the person and the geographic location. In traditional general-domain named-entity hierarchies and schemes, role names are not considered named entities. However, previous work has demonstrated that role names are a valuable piece of information to be retrieved for the Digital Humanities community. After all, role names allow to establish and contextualize relationships between people and their social position, their lineage, the places to which they belong, etc. See as an example the work developed by Murray (2017), where relationship dynamics from heterogeneous types of Medieval texts are analyzed focusing on tensions between family members, including the study of domestic violence. Therefore, although information related to the social role, lineage, nobility title or geographical origin of the person being mentioned is not usually encoded in traditional NER schemes, it should not be overlooked when working on the medieval domain. Furthemore, role names play a central role in medieval documents: kinship (and family relationships in general) are a fundamental identifier on legal documents (for example, when it involves nobility titles or inheritance); likewise, professions and positions become especially relevant in political documents. In fact, these roles and relationships are the usual way (and some times even the only way) in which certain people are referred to in medieval texts, as it is not the person itself that is central, but the institution or geopolitical entity that their position or title represents: both in legal and literary works, it is often the case that a certain person is exclusively identified through their social role name (e.g. *the prior*, *the king*). As a result, roles in medieval documents meet the defining criteria of referential unicity, referential autonomy, denominational stability and referential relativity established by Fort teal. (2009) for named entities. On the basis of the above, role names will be considered named entities in this annotation scheme and therefore annotated as such.
- **Miscellanea** covers any other named entity that does not fit in any of the previous categories and that may be useful to historical research. Some examples of miscellaneous named entities that can be found in medieval documents are important objects that were significant enough within the medieval society to bear a proper name of their own, like sword names (e.g. *Excalibur*, *Tizona*, *Colada*), horses names (*Bucéfalo* or *Bucephalus*, the horse of Alexander the Great), book names (*the Gospels*) or chants and prayers (*Hail Mary*). This category includes every entity that is personified in the text with a proper noun. It should be considered that the act of naming weapons, animals or religious artifacts comes from the historical or literary role that those named entities fulfill, from a cultural or narrative point of view. In any case, future refinements of this category are expected after evaluating with a group of experts the research questions that this type of information might help address.

**Table 1:** Examples of named entities for every category: general-domain vs medieval world

Named-entity category	Medieval example	General-domain example
Person name	*Alexander the Great, Aphrodite*	*Elvis Presley, Hillary Clinton*
Location name	*Kingdom of León, Burgos Cathedral*	*New York City, Brooklyn Bridge*
Organization name	*Order of Saint James, the Trojans*	*The Washington Post, The Beatles*
Role name	*The King of Castilla, the Pope*	–
Miscellanea	*Babieca, Tizona*	*Nessie*

## Annotation scheme: format and tagset

In this section we describe the annotation scheme: first, the encoding format is described; second, a comprehensive description of the tagset itself is provided.

### Named-entity annotation format

Annotation coding formats should be as clear, reusable, unambiguous, semantically adequate, consistent, expressive, platform-independent and self-explanatory as possible (Ide and Romary 2004; Stede and Huang 2012).

In addition to these general criteria, when dealing with named-entity annotation we should bear in mind the nature and scope of the texts that we aim to annotate: after all, named-entity annotation for medieval manuscripts (or any other historical document) concerns mainly scholars working on the field of cultural heritage, social sciences and the humanities, fields which tend to be quite apart from the NLP community and its developments and standards. Fortunately, there is a common ground that enables the exchange between these two apparently unrelated areas of knowledge: the Text Encoding Initiative (Text 2008).

**XML-TEI markup scheme** has become the prevailing standard for the representation of texts in digital form among digital humanists (Pierazzo 2016). In fact, the suitability of XML-TEI format for NER purposes was pointed out as early as MUC-7 guidelines (Chinchor 1998), although the proposal never gained full traction within the NLP community. XML-TEI meets the requirements in terms of annotation (re)usability and generalization, and its tagset is wide, flexible and expressive enough to account accurately for the historic NER categories within the medieval textual tradition.

Furthermore, XML-TEI format allows to limit the annotation tagset to a few common general tags while adding as many specificities and subcategorization as needed in the attributes. This feature is particularly interesting when addressing a highly specific field like the medieval domain, as it allows the annotation to be as comprehensive as needed while avoiding producing too high a number of different categories in the annotation scheme, which can cause lower interannotator agreement levels (Bayerl et al. 2003). This combination of coarse-grained tags with fine-grained attributes enables a double level where XML-TEI standard tags coexist with more granular attributes, ensuring an interoperable, reliable and yet enriched and comprehensive annotation.

Likewise, XML format facilitates element nesting, which can be extremely useful when annotating medieval entities that contain other entities within, which is usually the case when annotating nobility titles. Shall the annotator encounter a nobility title name such as *king of Castilla and Aragón*, XML nesting will allow annotating both the main entity (the role name *king of Castilla and Aragón*) and the geographical locations linked to the nobility title (*Castilla* and *Aragón*), if a more detailed annotation is preferred.

Due to its interoperability, semantic expressiveness, nesting flexibility and popularity within the Digital Humanities community, we consider XML-TEI the ideal format for medieval named-entity annotation.

### Named-entity annotation scheme

We will now list the proposed XML-TEI tags for every named-entity category described in Sect. 2.2, along with the attributes and values that should be added to cover all subcategories. Every XML-TEI tag will be illustrated with examples extracted from our Spanish medieval corpus, which will be described in Sect. 5. For the sake of clarity, either translations or equivalent examples in English will also be provided. It should be noted that some of the attribute values that we propose have no documented previous use and are (to our knowledge) original contributions of this work. These undocumented attribute values can be distinguished from the TEI-complying attribute values because the former ones are marked using an underscore (type = ”_value”). The proposed annotation scheme for medieval named-entities is inspired by the MUC-7 and ACE guidelines (Chinchor 1998; Linguistic 2005) and by Sekine’s Extended Named Entity Hierarchy. Sekine (2003). See Table 2 for a comparison between the proposed annotation scheme and Chinchor (1998), Linguistic (2005) and Sekine (2003).

#### Person names

**First names, surnames and family names** will be annotated using the XML-TEI tag <persName>. Although TEI offers specific tags for first names and surnames, this annotation scheme makes no distinction between first names and surnames, as this distinction becomes particularly fuzzy when it comes to medieval and historical names. The entire name shall be annotated using one <persName> tag only.
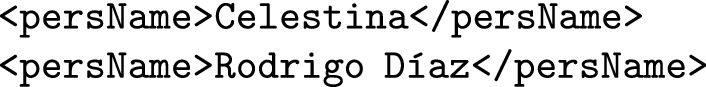


**Nicknames and aliases** have two different annotation approaches, depending on whether the nickname appears as part of the official name or *in lieu* of it. The reason behind this distinction is to comply with the TEI guidelines^3^:

1. If the nickname appears as part of the official name, it will be annotated within the <persName> tag using the nested TEI tag <addName>. This will enable nickname retrieval and classification. The <addName> tag should also be used to tag epithets (such as *Alexander the Great* or *Pedro el Cruel*) and regnal numbers (*Alfonso VI*).

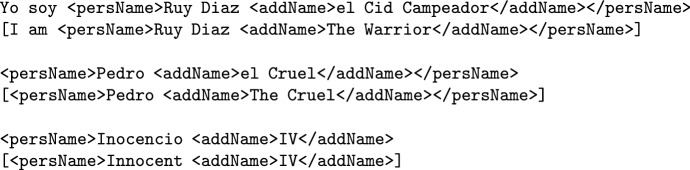

2. Lone nicknames (i.e. the nickname appears by itself, with no official name around) will be annotated using the <persName> tag followed by the TEI attribute type = "nickname", which indicates that the entity is not the real name of the person, but an alias.

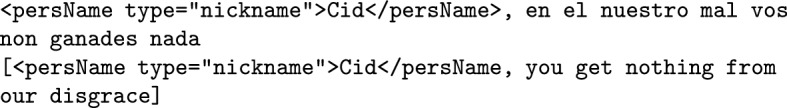


Lone nicknames were annotated differently from additional name components in order to highlight that they are consolidated nicknames that function as official names.

**Deities and divine figures** (such as gods and saints) will also be considered <persName>, but in order to distinguish them from normal person entities they will bear the attribute type="_deity" within the tag. 
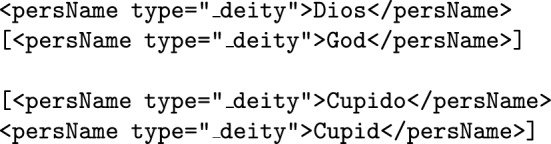


**Biblical nicknames and other divines aliases** (such as *Christ*, *Lord*, *King of Kings*...) are frequent in medieval literature and work *de facto* as indirect names for God and other divine figures. The nature of these names combines notions related to two categories in our annotation scheme, i.e. deity names and nicknames: we formalize deity names with a type="_deity" attribute, whereas we tag nicknames via a type="nickname" attribute. Since biblical nicknames and other divine aliases have a commonality with both deity names and nicknames, we propose an additional type combining both: type="_nickname_deity". This attribute enables the retrieval of all religious nicknames that appear in a given text, which can be of great use when working with biblical texts and other religious sources. 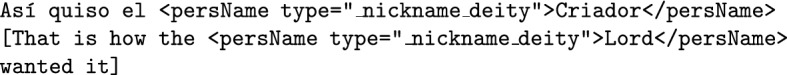


#### Location names

**Geopolitical units** (such as countries, cities, towns, villages, regions, etc) will be annotated under the TEI tag <placeName>. 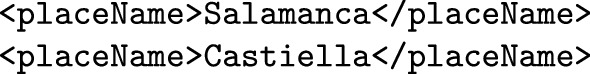


**Facilities** such as buildings or monuments that refer to a specific location (castles, monasteries, bridges, etc) should be annotated using the TEI tag <placeName> followed by the attribute type="_facility". 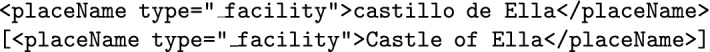


**Landforms and other natural geographical features** (such as rivers, mountains, oceans, etc) should be annotated under the TEI tag <geogName>. 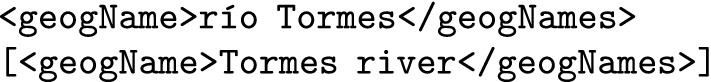 This approach is based on the hierarchy defined by Nadeau and Sekine (2007), as well as the TEI guidelines^4^. We are aware of the difficulties that making the difference between human-defined places and geographical accidents poses to automatic processing. However, keeping this difference on the annotation scheme can provide new knowledge to the study of historical place names and on how they came into existence in the first place.

**Religious, mythological and fictitious places** (such as *Garden of Eden*, *Paradise* or *Styx river*) will also be considered <placeName> or <geogName> (where appropriate) but will be followed by the attribute type="_mythological". This attribute facilitates making the difference between locations that can be tracked on a map and those that cannot. 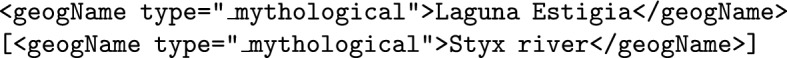


#### Organization names

**Organizations, associations, institutions and other groups of people** (such as religious orders, armies or governmental institutions) should be annotated under the TEI tag <orgName>. 
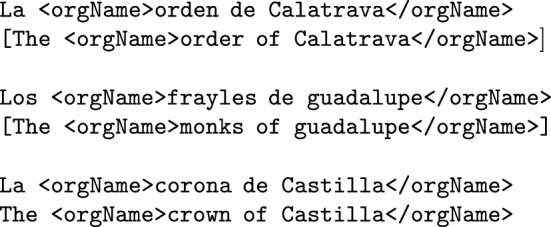


**Peoples, communities and any coherent group of people that share a sense of unity or cause** like political organizations or ethnic groups (such as *the Greeks*, *the Trojans*, *the Moors*, *the Jews* or *the Christians*) shall be annotated as <orgName>, following (Sekine 2003). This <orgName> tag will be followed by the attribute type = "_collective". Likewise, other groups of people that function as a unit (such as *the Three Wise Men* or *the Twelve Apostles*) will also be annotated under the <orgName type = "_collective">. 
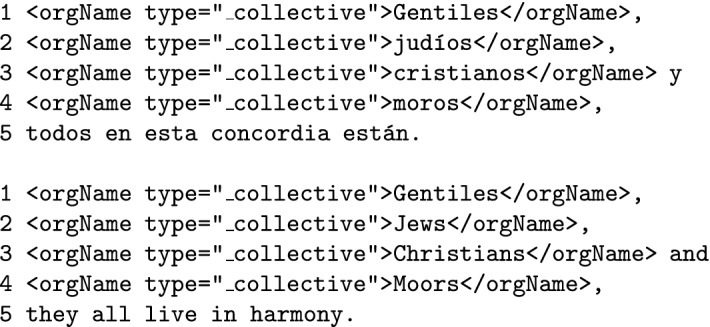


#### Role names

**Positions, nobility titles and professions** (such as *Queen of Castile*, *Bishop of Oviedo* or *king* appearing in isolation) will be annotated with the TEI tag <roleName>. 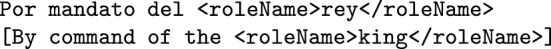 The <roleName> tag can be nested within person names or contain other nested tag (like locations or organizations):] 
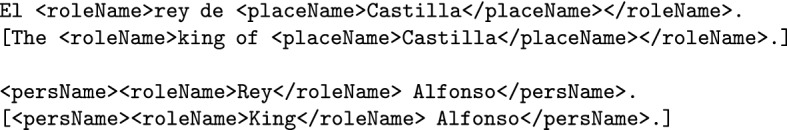


**Saints, angels, archangels, prophets and other religious roles** (which usually precede a person name) should also be annotated as role names: 
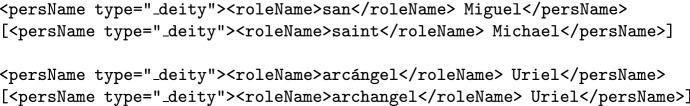


**Legitimacy expressions and other divine right formalisms** (such as *by the Grace of God* or *por la gracia de Dios*) usually appear next to nobility titles and political positions in medieval documents. In fact, this kind of expressions work as a formal prelude for certain positions and titles and therefore they can be considered part of the role name itself (as they express the legitimacy that provides the ruler with divine right to hold that title). These expressions should be annotated with <addName> along with the attribute type="_legitimacy". The <addName> tag should be nested within the appropriate <rolename> tag. 
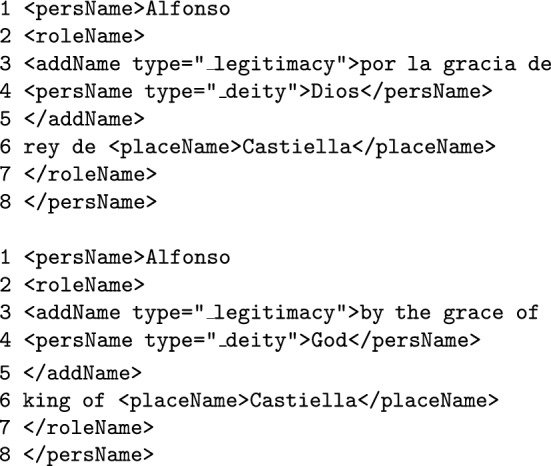


Legitimacy expressions are commonly used in historical and legal texts, and they legitimate the roles exhibited while naming a person. The expressions are not an entity in themselves, but they qualify a name, providing historical legitimization.

**Family relationships** (such as *mother*, *daughter*, *father*...) should also be annotated with the TEI tag <roleName> using the specific attribute type="_family".
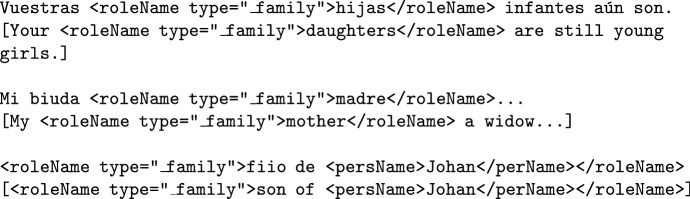


**Honorifics** (such as *mister*, *sir*, *lady*, *don*, *señor*, *doña*, etc) should also be considered role names and be annotated as nested tags within the appropriate <persName> tag. Honorifics will be annotated as <roleNames> with the TEI attribute type="honorific" attached.
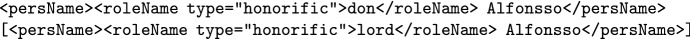


#### Miscellanea

Any other name that should be annotated as a named entity but that does not fit in the previous categories can be tagged under the miscellaneous TEI tag <name>. This tag can be used by annotators to annotate any additional entities that could bear historical interest but that do not fit in any of the previous categories. 
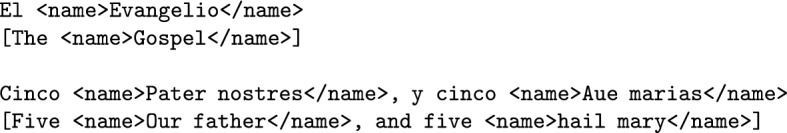

**Table 2:** Annotation scheme comparison

Named-entity category	Prototypical example	Real example from Spanish medieval corpus	MUC-7 guidelines (Chinchor 1998)
Person	*Elizabeth II of England, John Fitzgerald Kennedy*	*Guayo Julio Cesar* (*Julius Caesar*)	PERSON
Fictional character	*Mickey Mouse, Popeye*	*Celestina*	PERSON
Gods and deities	*Zeus*	*Fortuna, Venus*	PERSON
Nicknames	*Mr. Fix-It*	*El Cid, El Campeador* (*The Warrior*)	PERSON
Animals (with proper name)	*Nessie, Morris the cat*	*Babieca, Bucéfalo* (*Bucephalus*)	PERSON
Organizations, institutions, corporations	*Massachusetts Institute of Technology*	*Orden de Santyago* (*Order of Santiago*)	ORGANIZATION
Cities, towns, regions, countries	*New York City, China*	*Roma, Mesopotamia, Asia*	LOCATION
Landforms	*Mississippi river*	*Río Tormes, los Alpes* (*Tormes river, the Alps*)	LOCATION
Buildings, monuments	*Eiffel Tower*	*Castillo de Ella* (*Ella castle*)	ORGANIZATION
Honorifics	*Lord, sir, miss*	*Don, sennora, maese*	No
Group of people regarded as a single unit	*The Beatles*	*Los moros, los santos* (*the Moors, the Saints*)	No
Positions and nobility titles	*President of USA*	*Rey de Castiella* (*King of Castilla*)	No
Family relationships	*The lieutenant’s daughter*	*Fiio del Rey Iohan* (*King Johan’s son*)	No
Legitimacy expressions	-	*Por la gracia de Dios* (*by the grace of God*)	No

Named-entity category	ACE guidelines (Linguistic 2005)	Extended named entity hierarchy (Sekine 2003)	TEI annotation scheme for medieval named entities
Person	Person (PER)	Person	$\texttt {<persName>}$
Fictional character	Person (PER)	Product: Character	$\texttt {<persName>}$
Gods and deities	Person (PER)	God	$\texttt {<persName type="\_deity">}$
Nicknames	Person (PER)	Person	$\texttt {<persName type="nickname">} or \texttt {<addName>}$
Animals (with proper name)	No	Name_Other	$\texttt {<name>}$
Organizations, institutions, corporations	Organization (ORG)	Organization	$\texttt {<orgName>}$
Cities, towns, regions, countries	Geo-political Entity (GPE)	Location	$\texttt {<placeName>}$
Landforms	Location (LOC)	Location: Geological_Region	$\texttt {<geogName>}$
Buildings, monuments	Facility (FAC)	Facility	$\texttt {<placeName type="\_facility">}$
Honorifics	No	Product: Title: Title_Other	$\texttt {<roleName type="honorific">}$
Group of people regarded as a single unit	Geo-political Entity (GPE.PER) or PER.Group	Organization	$\texttt {<orgName type="\_collective">}$
Positions and nobility titles	No	Product: Title: Position_Vocation	$\texttt {<roleName>}$
Family relationships	No	No	$\texttt {<roleName type="\_family">}$
Legitimacy expressions	No	No	$\texttt {<addName type="\_legitimacy">}$

## Annotation guidelines

In this subsection we describe the guidelines to follow and address the most common unclear cases that can be encountered when applying the previous annotation scheme to a medieval corpus.

### Medieval aliases

As it was mentioned on the previous section, nicknames are so common in medieval texts that they even become the standard person name on official documents; accordingly, nicknames have their own tag format within the annotation scheme. However, only those nicknames that are fully established and work *de facto* as person names should be taken into account. In other words, only nicknames that answer the question *What is the name of this person?* should be annotated. Poetic phrases that refer indirectly to people or characters on literary medieval texts and that are not the established name of the person (such as *the one from Troy* to refer to *Paris*), will not be considered as real person names for our annotation purposes.

### Designators and specifiers

Geopolitical names are frequently preceded by a locative designator or specifier (i.e. structures such as *kingdom of* or *town of*). These designator are usually a part of the official medieval name itself and therefore should be annotated as part of the named entity: 
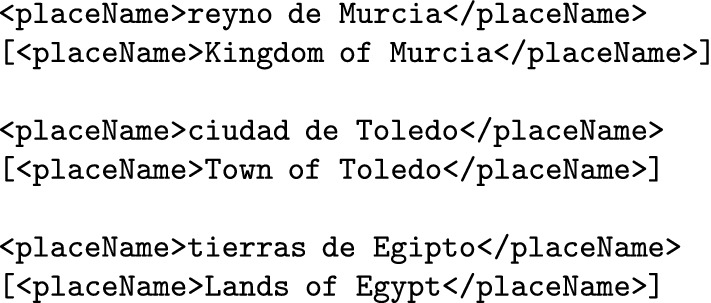


### Nested tags

Person names, location names and role names may have other entities within them. For example, role names and person names can have location names within (*Elizabeth of England*, *Duke of Edinburgh*) or be nested within other named entities. In these cases, we will seize the nesting possibilities of XML-TEI and applied nested tags when necessary. This granularity allows a more complete annotation and therefore ensures a more informative entity retrieval. Some examples of nested tags follow: 
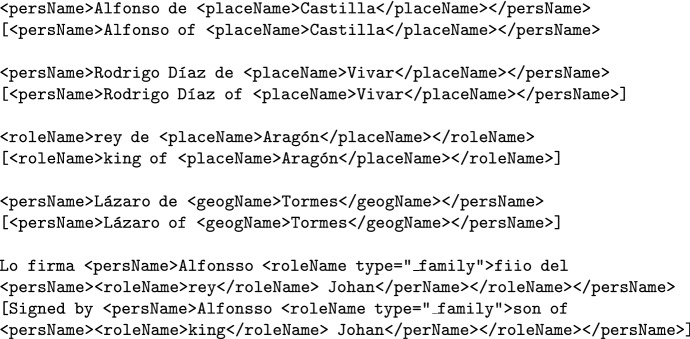


### Combined named entities (personName+roleName+placeName)

In medieval legal documents, complex named entities that refer to a person may combine first names, nobility titles, positions and kinship (*Alfonso, king of Castilla*) meaning that several persName, roleName and placeName can be found. As a rule of thumb, whenever a person name is explicit $(\texttt {<persName>})$, that will be the main and most external tag, while the rest of tags $(\texttt {<roleName>}, \texttt {<placeName>}$, etc) will be nested inside the main $\texttt {<persName>}$ tag. 
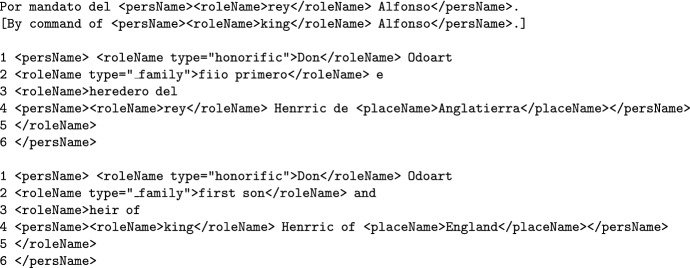


### Ambiguity between family names and birthplaces

Medieval person names can have a second element that appears after the first name and is preceeded by the preposition *de* (“of”, “from”). This second element resembles a surname but could either be a family name (and therefore should simply be annotated as part of the $\texttt {<persName>}$, as in example 1) or a birthplace working as a patronymic (and therefore should be annotated as $\texttt {<placeName>}$, as in example 2):
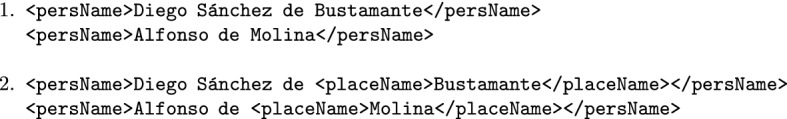
 In order to ensure a precise annotation, the most informative solution for these cases requires looking these names up in historical records and checking the existence of the town or region in question at the time when the character was alive. If no solid information backs up the hypothesis of that name being in fact the birthplace of the person, it should simply be annotated as part of the name (as was done in example 1).

### Kingdoms: place names or surnames?

Kings and queens tend to have particularly long and complex names that combine titles, names and kingdoms, especially on legal documents. This complexity can make the annotation process tricky.

Let’s take the case of *rey Alfonso de Castilla* (*King Alfonso of Castilla*), that is apparently composed of the person name *Alfonso* and the role name *rey de Castilla* (*king of Castilla*). At first sight, annotating *king of Castilla* as an entire $\texttt {<roleName>}$ would seem like the most natural approach. But in that case the annotator would have to choose between two rather unsatisfactory options: either making a discontinuous tag between *rey* (*king*) and *de Castilla* (*of Castilla*) in order to leave *Alfonso* out of the $\texttt {<roleName>}$ tag, or consider everything as a $\texttt {<roleName>}$ (including person name *Alfonso*), which would be against the guideline that states that whenever a person name is explicit, $\texttt {<persName>}$ should be the main and most external tag (see Sect. 4.4). Our proposed solution for this type of situation is to apply the $\texttt {<roleName>}$ tag only to *rey* (*king*), leaving *Castilla* as part of the $\texttt {<persName>}$ tag. This decision implies that *Castilla* will not be directly attached to the role name *king*, but would work somehow as a kind of surname. 
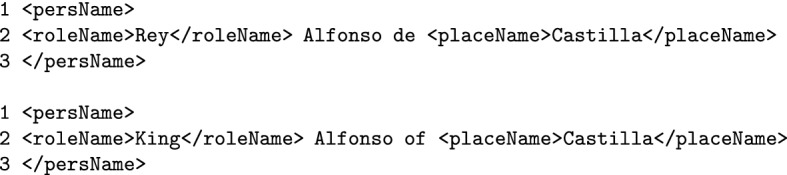
 At first sight, this workaround can seem quite unorthodox, but it solves several issues:

1. It avoids discontinuous tags.
2. It complies with the guideline that states that whenever an entity refers to a person and the person name is present, $\texttt {<persName>}$ should be the main tag (Sect. 4.4).
3. It is consistent with other NER annotating systems (see example *Elizabeth II of the United Kingdom* under Person category in Sekine (2003)).

### Organization names vs place names: metonymies

A common problem that general-domain NER faces is how to deal with metonymy (Poibeau 2006). Metonymy is the linguistic phenomenon in which a part of an object or its location is used to refer to the entire object. A classic metonymy example is when the name of a country or region is used to refer to its population or government (Markert et al. 2002).

The medieval domain is not an exception to this phenomenon. In medieval documents, metonymy occurs frequently when a place name or facility name (such as a country, a monastery, a city, etc) is used to refer to an institution, in other words, the name of a place represents both a physical location and an administrative entity. When annotating this type of named entities the annotator can be unsure on whether these entities should be annotated literally (i.e. under $\texttt {<placeName>})$ or figuratively (i.e. as $\texttt {<orgName>})$.

For these borderlines cases, the TEI guidelines criteria should be followed, Text (2008): if the physical location corresponds to a district, region, locality or any other geopolitical unit (such as cities, countries, etc) it should be annotated as $\texttt {<placeName>}$. If the named entity is a different type of location (such as a building, institution, etc), the tag $\texttt {<orgName>}$ will be preferred.^5^

## Annotation process and results

The annotation guidelines we have just presented were implemented on a corpus of medieval Spanish. In this section we describe the composition of the annotated corpus and we discuss the obtained results.

### Composition of the corpus

The annotated corpus is a compilation of digitized manuscripts in medieval Spanish edited by the Hispanic Seminary of Medieval Studies^6^ (Jover 2015). The corpus contained 26,200 words and 2,054 named entities. The annotated texts were paleographic editions, which means that the texts reflected the exact writing that appeared on the original text source, including errors, omissions and non-standardized spelling. The manuscripts ranged from the 13th century to the 16th century and included chronicles, legal documents and literary texts (narrative, poetry and theatre).

With regard to the genre distribution of the corpus, the majority of the corpus contained literary texts (70%), including some of the most prominent literary works in Spanish from that time period, such as *El Cantar de Mio Cid*, *Libro de Buen Amor* or *La Celestina*. In this genre, *El Cantar de Mio Cid* deserves a special mention, since a great amount of entities from all categories in our annotation scheme were identified on it. Legal texts represent 20% of the corpus and were particularly rich in complex person names and nobility titles. Historiographical texts (such as chronicles) account for 10% of the corpus and they were crucial to test the annotation of historical people, places, organizations (such as religious orders) and names associated to divinities and saints.

### Results and discussion

The corpus was annotated twice by two independent annotators that were trained on linguistic annotation and were provided with the presented annotation guidelines prior to the annotation process^7^. In order to establish the coherence between both annotations, the kappa coefficient was measured (Carletta 1996; Artstein and Poesio 2008). The resulting inter-annotation agreement was 0.802 (N=2054, K=2). This can be seen as an indication of the reliability of the annotation scheme, as Landis and Koch (1977) and Krippendorff (1980) consider a coefficient above 0.8 as indicating reliable annotations.

Regarding the annotation disagreements, 258 issues out of 2,132 annotations were identified (12.10% of all the annotations). The source of disagreement may be classified into three main groups: choice of markable, tag selection and tag swap.

1. **Choice of markable** The choice of markable disagreements are text spans that were tagged by one annotator but not the other. This was by far the most frequent source of disagreement, accounting for 86% of all disagreements (226 out of 258 total disagreements: 110 issues attributable to annotator 1; 116 attributable to annotator 2). These disagreements may reflect different perceptions by annotators of nuances in the texts or in the medieval reality (such as annotating a given location as placeName or a given relation or position as roleName). See Table 3 for a quantification of the choice of markable disagreements between annotators grouped by tag.
2. **Tag selection** Tag selection disagreements concern spans that were identified as named entities by both annotators but were annotated using different tags. This type of disagreement accounts for 8,5% of all disagreements (22 issues out of 258 total disagreements). Half of these differences were related to the selection of either the <orgName> tag or the <roleName> tag, specifically when annotating certain religious orders. These errors arose from the subtlety of considering the mention of a religious order as a reference to the members that belong to that religious order (a role) or a reference to the order itself (an organization): 
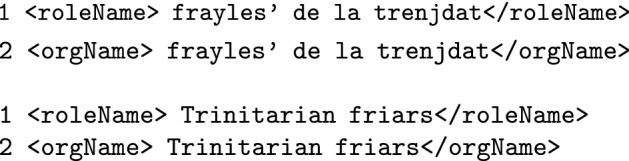

**Table 3:** Annotation Error Summary for the error type ‘Choice of markable’

Tag	Annotator 1	Annotator 2
roleName	34	71
placeName	7	10
orgName	27	5
name	9	9
persName	27	20
addName	3	1
geogName	3	0
Total	110	116
Percentage from total errors	42%	44%
Percentage from total annotations	5.16%	5.44%
3. **Tag swap** Tag swap disagreements consists of human errors derived from the application of the annotation scheme to complex entities and consist mostly of nesting errors (see example below). This source of disagreement accounts for 6.5% of all disagreements (17 cases out of 258 total errors). 
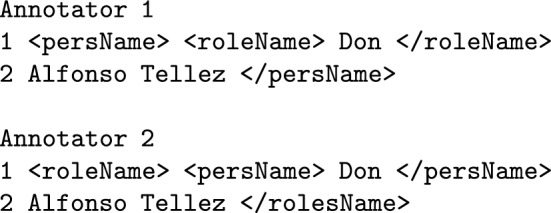

## Conclusions and future work

We have described a NER annotation scheme for medieval texts intended to capture the nature of medieval documents. The proposed category set is an extension of traditional journalistic NER hierarchies and takes into account the key role that nobility titles, positions and family relations played in medieval society (and, consequently, on medieval documents). The proposed annotation scheme tagset is encoded in XML-TEI to ensure interoperability and facilitate its use and exploitation among humanist researchers. Finally, explicit annotation guidelines were introduced and discussed in order to ensure a coherent and reliable annotation and clarify the possible obscure cases that may arise from the special nature of medieval reality.

The presented annotation scheme and guidelines were implemented and tested on a corpus of medieval Spanish, producing an inter-annotator agreement of 0.8 (kappa), which can be considered a solid annotation. Therefore the proposed annotation scheme can be considered a reliable scheme if applied to future NER tasks that involve the annotation of medieval manuscripts and other historical documents.

The enriched annotation we have presented provides valuable source materials for researchers in the Digital Humanities. The annotation of medieval role names is, to the best of our knowledge, a novel contribution from this work that provides new and valuable information in terms of historical contextualization of a document: although role name positions are traditionally not considered named entities in off-the-shelf named-entity hierarchies, the annotation of role names in medieval documents allows to establish the relation between people, places and lineages mentioned in a text, enhancing the contextualization of the document, the identification of relation between entities and the setting of a chronological framework for the text. Thanks to the annotation of role names, the tool HisMeTag (which implements the annotation system we have just presented) can provide such relation graphs by implementing the nested tagging as dependency trees.

In terms of future work, it would be interesting to assess the suitability of the presented scheme on documents from other historical periods of time (from the Renaissance onwards). In addition, an interesting avenue for future work would be to address the coverage of the presented scheme in other historical named entities and to explore the possibility of subdividing the general name category into more fine-grained categories related to specific subdomains.

From a wider perspective, medieval documents are a rich source of valuable information that await being exploited in terms of natural language processing. Further work is needed towards the textual exploitation of information contained on historical archives, a task that requires the collaboration between both the NLP and Digital Humanities communities.

## Footnotes

1 See http://commons.pelagios.org/.

2 For a report about the annotation of place names in literary texts in a TEI environment see Frontini et al. (2016).

3 “Additional name contains an additional name component, such as a nickname, epithet, or alias, or any other descriptive phrase used within a personal name”. See https://www.tei-c.org/release/doc/tei-p5-doc/es/html/ref-addName.html.

4 “Geographical name identifies a name associated with some geographical feature such as Windrush Valley or Mount Sinai". See https://www.tei-c.org/release/doc/tei-p5-doc/en/html/ref-geogName.html.

5 It should be noted that this guideline is compliant with TEI, but it diverts from ACE guidelines, where names of places are annotated as organizations when they refer to sports team, etc.

6 http://www.hispanicseminary.org/.

7 Annotation guidelines in Spanish available at http://postdata.linhd.uned.es/guia-de-anotacion-de-entidades-nombradas-medievales/.
